# Supplementary material for: Analysis of PPARγ Signaling Activity in Psoriasis
Source: Int J Mol Sci. 2021 Aug 10;22(16):8603. doi: 10.3390/ijms22168603 (PMC8395241; doi:10.3390/ijms22168603)
Supplement: Supplementary file 1 [file ijms-22-08603-s001.zip › Supplemental materials_Analysis of PPARg signaling activity in psoriasis/Pathway models/Models images and html files/Interleukin-17 and interleukin-22 signaling in psoriasis/471.html]

AKT1


# Protein AKT1

|  |  |
| --- | --- |
| URN | urn:agi-llid:207 |
| Total Entities | 0 |
| Connectivity | 19439 |
| Name | AKT1 |
| Description | AKT serine/threonine kinase 1 |
| Notes | The serine-threonine protein kinase encoded by the AKT1 gene is catalytically inactive in serum-starved primary and immortalized fibroblasts. AKT1 and the related AKT2 are activated by platelet-derived growth factor. The activation is rapid and specific, and it is abrogated by mutations in the pleckstrin homology domain of AKT1. It was shown that the activation occurs through phosphatidylinositol 3-kinase. In the developing nervous system AKT is a critical mediator of growth factor-induced neuronal survival. Survival factors can suppress apoptosis in a transcription-independent manner by activating the serine/threonine kinase AKT1, which then phosphorylates and inactivates components of the apoptotic machinery. Mutations in this gene have been associated with the Proteus syndrome. Multiple alternatively spliced transcript variants have been found for this gene. [provided by RefSeq, Jul 2011] |
| Primary Cell Localization | Cytoplasm |
| Class | Protein kinase |
| ObjectType | Protein |

---

|  |  |
| --- | --- |
| Pathway | Proteins Involved in Stem Cell Exhaustion in Aging |
|  | Osteoarthritis |
|  | Androgen Deficiency in Male Obesity |
|  | Oncogene Induced Cellular Senescence |
|  | MTHFR Activity Regulation |
|  | DNA Persistent Repair Inhibits mTOR Signaling |
|  | mTOR Signaling Activation by Fatty Acids and Glucose |
|  | mTOR Signaling Activation by Amino Acids |
|  | mTOR Signaling |
|  | mTOR Signaling Hyperfunction |
|  | Telomer Attrition Triggers Genomic Instability |
|  | Insulin Influence on Protein Synthesis |
|  | Insulin Influence on Glycogenesis |
|  | Insulin Influence on Lipogenesis |
|  | Dopamine Receptors Signaling |
|  | Thyroid Hormones Common non-Genomic Effects in Hyperthyroidism |
|  | Insulin Signaling |
|  | Thrombopoietin Receptors Signaling in Platelet Maturation |
|  | Androgens in Adipocyte Activation |
|  | LH and FSH Signaling and Steroidogenesis |
|  | Toll-like Receptors Act through MYD88-TIRAP Signaling |
|  | T-Cell Dependent B-Cell Activation |
|  | T-Cell Independent B-Cell Activation |
|  | Natural Killer Cell Activation through C-Type Lectin-like Receptors |
|  | T-Cell Maturation (Hypothesis) |
|  | Peripheral T-Cell Tolerance |
|  | T-Cell Positive Selection and Neglect Induced Death |
|  | T-Cell Central Tolerance |
|  | CD8+ T-Cell Activation |
|  | Th1-Cell Differentiation |
|  | Treg-Cell Differentiation |
|  | Th2-Cell Differentiation |
|  | T-Cell Receptor Signaling |
|  | Th17-Cell Differentiation |
|  | AHR Signaling in Tr1 Cells Function |
|  | CXC Chemokine Receptor Signaling |
|  | CC Chemokine Receptor Signaling |
|  | Plasmin Effects in Inflammation |
|  | TAM Receptors in Platelet Aggregation |
|  | Platelet Activation via Adhesion Molecules |
|  | Proplatelet Maturation |
|  | Bradykinin Effects in Inflammation |
|  | Platelet Activation via GPCR Signaling |
|  | Toll-like Receptors in Sterile Inflammation |
|  | Toll-like Receptor Independent Sterile Inflammation |
|  | Eicosanoids in Myeloid Cell Activation |
|  | Histamine Receptor H3 Signaling in Neurons |
|  | Neutrophil Activation via LTB4/Polyisoprenyl Phosphate Signaling |
|  | Basophil Activation |
|  | Eosinophil Survival by Cytokine Signaling |
|  | Eosinophil Activation |
|  | Mast-Cell Activation via IgE Signaling |
|  | Mast-Cell Activation without Degranulation |
|  | Mast-Cell Activation without Degranulation through KIT/F2R Signaling |
|  | Mast-Cells De Novo Synthesized Mediators via IgE Independent Signaling |
|  | VEGFA/NOTCH1/WNT Cross-talk in Blood Vessel Sprouting and Branching in Cancer |
|  | Vascular Endothelial Cell Activation by NO |
|  | Vascular Endothelial Cell Activation by Cytokines |
|  | Vascular Endothelial Cell Activation by Blood Coagulation Factors |
|  | Vascular Endothelial Cell Activation by Growth Factors |
|  | Vascular Endothelial Cell Permeability Activation |
|  | Adipokines Production by Adipocyte |
|  | Lipolysis Regulation in Adipocyte |
|  | Lipogenesis Regulation in Adipocyte |
|  | Synaptic Potentiation by PKMZ in LTP Maintenance - Active State |
|  | Nociception Expression Targets Signaling |
|  | Sodium Flux Regulation |
|  | Vascular Motility |
|  | BDKRB1/2 -> Vasodilation |
|  | BDKRB1/2 -> Interleukins Production |
|  | DRD2 -> TRPC1 Transcription |
|  | GALR1/2/3 -> POMC/NPY Production |
|  | PTGER2/3 -> Vascular Motility |
|  | TACR1 -> TNF/IL6/IL8 Production |
|  | Leptin -> NO Production/Vasodilation |
|  | Kidney Cells Cilia Organization |
|  | Apoptosis |
|  | G1/S Phase Transition |
|  | Cell Cycle Overiew |
|  | G0/G1 Phase Transition |
|  | G2/M Phase Transition |
|  | Tight Junction Assembly (Occludin) |
|  | Adherens Junction Assembly (Cadherins) |
|  | Actin Cytoskeleton |
|  | Telomere Maintenance |
|  | Axonal Transport |
|  | RAGE/AGER and S100 Proteins in Cardiovascular Injury |
|  | Th1-Cells Activation and Proliferation in Atherosclerosis |
|  | Proteins Involved in Dilated Cardiomyopathy |
|  | Proteins Involved in Hypertrophic Cardiomyopathy |
|  | Cardiomyocyte Hypertrophy |
|  | Hypertrophic Cardiomyopathy |
|  | Proteins Involved in Arterial Hypertension |
|  | Endothelin Signaling in Arterial Hypertension |
|  | Arterial Hypertension |
|  | Endothelial Cell Dysfunction in Arterial Hypertension |
|  | Smooth Muscle Cell Dysfunction in Arterial Hypertension |
|  | TRPM4/6/7/8 Signaling in Arterial Hypertension (Hypothesis) |
|  | BMP2 and WNT Signaling in Pulmonary Artery Smooth Muscle Cells |
|  | Myocardial Remodeling |
|  | Myocardial Infarction |
|  | Proteins Involved in Myocardial Ischemia |
|  | Preconditioning Ischemia |
|  | Lymphocyte Mediated Myocardial Injury in Myocarditis |
|  | Myocarditis, Acute Viral Overiew |
|  | Vascular Reactivity in Raynaud Disease |
|  | NO Synthesis in Early Vasospasm |
|  | Proteins Involved in Acne Vulgaris |
|  | Hyperseborrhea in Acne Vulgaris |
|  | Androgen Induced Sebocyte Hyperfunction |
|  | Androgens in Sebocyte Maturation |
|  | Atopic Dermatitis Onset |
|  | Proteins Involved in Ichthyosis |
|  | Melanoma |
|  | Apoptosis Block in Melanoma Cell |
|  | Dedifferentiation and Metastatic Progression in Melanoma |
|  | Proteins Involved in Melanoma |
|  | IFN-gamma/TNF-alpha Mediated Cell Proliferation in Psoriasis |
|  | IL22 Induces Keratinocyte Proliferation in Psoriasis |
|  | Proteins with Altered Expression in Psoriasis |
|  | Dendritic Cells Function in Psoriasis |
|  | Th0-Cell Aberrant Activation in Systemic Lupus Erythematosus |
|  | Th1-Cell Function in Systemic Lupus Erythematosus |
|  | B-Cell Activation in Systemic Lupus Erythematosus |
|  | Th2-Cell Function in Systemic Lupus Erythematosus |
|  | Acute Cytotoxic CD8+T-Cell Response against Melanocytes |
|  | T-Cell Cytotoxic Response against Melanocytes in Vitiligo |
|  | Humoral Immunity in Vitiligo |
|  | Treg-Cell Function in Diabetes Mellitus Type 1 |
|  | Natural Killer T-Cell Roles in Diabetes Mellitus Type 1 |
|  | Toll-like Receptors in beta-Cell |
|  | Macrophage Function in Diabetes Mellitus Type 1 |
|  | Treg-Cell Activation in Diabetes Mellitus |
|  | Proteins Involved in Diabetes Mellitus Type 2 |
|  | Donohue Syndrome Progression (Hypothesis) |
|  | Advanced Glycation End Products Pathway in Diabetic Microangiopathy |
|  | Protein Kinase C in Diabetic Microangiopathy |
|  | Peripheral Tissue Microangiopathy |
|  | Tubular Cell Dysfunction in Progressive Diabetic Nephropathy |
|  | Podocyte Dysfunction in Diabetic Nephropathy |
|  | Endothelial Cell Dysfunction in Early Diabetic Nephropathy |
|  | Proteins Involved in Diabetic Nephropathy |
|  | Mesangial Cell Dysfunction in Diabetic Nephropathy |
|  | Microangiopathy in Diabetic Neuropathy |
|  | Proliferative Diabetic Retinopathy |
|  | Glucocorticoids in Insulin Resistance in Skeletal Muscles |
|  | Adipokines and Cytokines in Insulin Resistance in Skeletal Muscles |
|  | AGE Related Insulin Resistance in Skeletal Muscles |
|  | Adiponectin in Insulin Resistance Prevention |
|  | Proteins Involved in Insulin Resistance |
|  | Insulin Resistance in Myocytes Induced by Obesity |
|  | Adiponectin Synthesis Declines in Insulin Resistance |
|  | Insulin Resistance in Hepatocytes |
|  | Proteins Involved in non-Alcoholic Fatty Liver Disease |
|  | Gluconeogenesis Impairment in non-Alcoholic Fatty Liver Disease |
|  | PNPLA3 in non-Alcoholic Fatty Liver Disease |
|  | Lipid Metabolism Impairement in non-Alcoholic Fatty Liver Disease |
|  | Leptin in Insulin Synthesis and Secretion |
|  | beta-Cell Destruction through Cytokines in Diabetes Mellitus |
|  | beta-Cell Mass Regulation |
|  | beta-Cell Function Inhibition by Ciclosporine and Tacrolimus (Rodent Model) |
|  | FOXO1 and SREBP-1C Role in beta-Cell Suppression (Rodent Model) |
|  | RET1 Mutation in Hyperparathyroidism |
|  | Bone Remodeling in Hyperthyroidism |
|  | Cardiovascular Effects in Hyperthyroidism |
|  | Thyroid Dysfunction |
|  | Graves Ophthalmopathy |
|  | Immune System Activation in Hyperthyroidism |
|  | Myocyte Dysfunction in Hypoparathyroidism |
|  | GLIS3 Targets in Thyroid Dysgenesis (Hypothesis) |
|  | Thyroid Stimulating Hormone (TSH) Resistance in Congenital Hypothyroidism |
|  | Overt Hypothyroidism, Primary |
|  | Thymic Follicular Hyperplasia |
|  | Adipocyte Hypertrophy and Hyperplasia |
|  | Adipokines Production by Adipocyte Impaired in Obesity |
|  | Proteins Involved in Obesity |
|  | CD4+T-Cell Response in Celiac Disease |
|  | Enterocyte Mediated Mucosal Damage in Celiac Disease |
|  | Proteins Involved in Colorectal Neoplasms |
|  | Cetuximab Resistance in Colorectal Cancer |
|  | Metastatic Colorectal Cancer |
|  | GUCY2C Signaling in Colorectal Cancer |
|  | OipA Signaling in Helicobacter Pylori Infection |
|  | Peutz-Jeghers Syndrome |
|  | Dendritic Cell Function in Ulcerative Colitis |
|  | Nicotine anti-Inflammatory Effect in Ulcerative Colitis |
|  | Proteins Involved in Ulcerative Colitis |
|  | Th2-Cell Function in Ulcerative Colitis |
|  | T-Cell Cytotoxic Response in Ulcerative Colitis |
|  | FLT3/KIT/MLL Signaling in Leukemia |
|  | Proteins Involved in Acute Myeloid Leukemia |
|  | Apoptosis Block in Acute Myeloid Leukemia Cell |
|  | RARA Signaling in Acute Myeloid Leukemia (M3) |
|  | Acute Myeloid Leukemia |
|  | B-Cell Acute Lymphoblastic Leukemia |
|  | Proteins Involved in B-Cell Chronic Lymphocytic Leukemia |
|  | B-Cell Chronic Lymphocytic Leukemia |
|  | Chronic Myeloid Leukemia |
|  | Proteins Involved in Chronic Myeloid Leukemia |
|  | Diffuse Large-B-Cell Lymphoma |
|  | Diffuse Large-B-Cell Lymphoma ABC Subtype |
|  | Proteins Involved in Diffuse Large-B-Cell Lymphoma |
|  | Lymphoma, GCB Subtype |
|  | Follicular Lymphoma |
|  | Hodgkin Lymphoma |
|  | Proteins Involved in Mantle Cell Lymphoma |
|  | Mantle Cell Lymphoma |
|  | Cell Cycle Dysregulation in Mantle Cell Lymphoma |
|  | Apoptosis of Mantle Cell Deregulation |
|  | mTOR/NF-kB/BCR Signaling Disregulation in Mantle Cell Lymphoma |
|  | Hedgehog Signaling in Mantle Cell Lymphoma |
|  | Mucosa-Associated Lymphoid Tissue (MALT) Lymphoma |
|  | Osteoclast Activation in Multiple Myeloma |
|  | IL6/IGF1/VEGFA Signaling in Multiple Myeloma |
|  | Multiple Myeloma |
|  | Proteins Involved in Multiple Myeloma |
|  | Proteins Involved in T-Cell Acute Lymphoblastic Leukemia |
|  | T-Cell Acute Lymphoblastic Leukemia |
|  | Proteins Involved in Hepatocellular Carcinoma |
|  | Hepatocellular Carcinoma |
|  | Growth Factor Signaling in Hepatocellular Carcinoma |
|  | Proteins Involved in Pancreatic Cancer |
|  | Pancreatic Ductal Carcinoma |
|  | Growth Factor Signaling in Pancreatic Neoplasms |
|  | Pancreatic Neuroendocrine Tumors |
|  | STK11 Signaling in Pancreatic Neoplasms |
|  | Il17 Signaling Related Neutrophilia in Asthma |
|  | Airway Smooth Muscle Cell High Rate Proliferation |
|  | IgE Induces Airway Smooth Muscle Cell Proliferation |
|  | Basophil Activation in Asthma |
|  | Mast-Cell Activation in Asthma |
|  | Mast-Cell Related Chemokines Signaling in Asthma |
|  | B-Cell Mediated IgE Production in Asthma |
|  | Th17-Cell Differentiation in Asthma |
|  | Th2-Cell Response in Asthma |
|  | Osteoclast Activation in Gout |
|  | Proteins Involved in Osteoarthritis |
|  | Proteins Involved in Polycystic Ovary Syndrome |
|  | Ovulation Block |
|  | SHBG and IGFBP1 Synthesis Decline in Polycystic Ovary Syndrome |
|  | Polycystic Ovary Syndrome |
|  | FSH Action in Polycystic Ovary Syndrome |
|  | Dendritic Cell Dysfunction in Psoriatic Arthritis |
|  | Th17-Cell and Th1 Immune Responsein Psoriatic Arthritis |
|  | Osteoclast Activation in Psoriatic Arthritis |
|  | Keratinocyte Activation in Psoriatic Arthritis |
|  | Osteoclast Activation in Rheumatoid Arthritis |
|  | Proteins Involved in Rheumatoid Arthritis |
|  | B-Cells Function in Systemic Scleroderma |
|  | Th2-Cells Function in Systemic Scleroderma |
|  | WHIM Syndrome |
|  | CD4+ T-Cell Function Decline in HIV |
|  | Macrophage Survival through CCR5 and CXCR4 Mediated Signaling |
|  | Toll-like Receptors (TLR) in Periodontitis |
|  | Diabetes Induced Periodontitis |
|  | Estrogen Effects on Pregnancy Gingivitis |
|  | IL1 Receptor Antagonist (IL1RN) Deficiency (DIRA) |
|  | TNF Receptor Associated Periodic Syndrome |
|  | Mevalonate Kinase Deficiency |
|  | Blau Syndrome |
|  | Familial Cold Autoinflammatory Syndrome 2 |
|  | Hereditary Hemorrhagic Telangiectasia |
|  | Cowden Syndrome |
|  | Glutamate Toxiicity Effects on Striatum Medium Spiny Neurons in Hantington's Disease |
|  | Huntingtin Mutant Protein Toxicity Effects |
|  | Proteins Involved in Huntington Disease |
|  | IL6 and Insulin Resistance in Muscular Dystrophies |
|  | IL6 Promotes Inflammation in Muscular Dystrophies |
|  | Ca2+ Overload in Muscular Dystrophies |
|  | Muscular Dystrophies |
|  | Dystrophin Glycoprotein Complex Signaling in Muscular Dystrophies |
|  | IGF1 Role in Muscle Hypertrophy |
|  | Myostatin-IGF1 Crosstalk in Skeletal Muscles in Muscular Dystrophies |
|  | Osteoblast Function Decline in Osteoporosis |
|  | Osteoclast Activation in Postmenopause |
|  | APP and Glutamate Signaling Cause Neuronal Dysfunction in Alzheimer Disease |
|  | Proteins Involved in Alzheimer's Disease |
|  | Ketogenic Diet Mechanism (Hypothesis) |
|  | Blood-Brain Barrier Disruption in Epileptiform Disorders |
|  | mTOR Hyperactivation after Status Epilepticus |
|  | Apoptosis and Immediate Early Gene Activation in Epileptiform Disorders |
|  | Glioblastoma, Primary |
|  | Proteins Involved in Glioblastoma |
|  | Glioblastoma, Secondary |
|  | Astrocytoma |
|  | Proteins Involved in Astrocytoma |
|  | Glioma Invasion Signaling |
|  | Proteins Involved in Glioma |
|  | Glioma Stem Cell Program Activation |
|  | Ependymoma |
|  | Medulloblastoma |
|  | Proteins Involved in Medulloblastoma |
|  | Vascular Endothelial Cell Vasodilation in Brain |
|  | Dopamine Action in CSD Progression |
|  | Postsynaptic Neuron Activation |
|  | nNOS (NOS1) and iNOS (NOS2) Activation via Glutamate |
|  | Growth Factor Signaling in Neuroblastoma |
|  | Neuroblastoma |
|  | ALK Associated Neuroblastoma |
|  | Sonic Hedgehog Signaling in Neuroblastoma |
|  | Proteins Involved in Neuroblastoma |
|  | Glutamate Role in Parkinson's Disease |
|  | Proteins Involved in Parkinson's Disease |
|  | Proteins Involved in Amyotrophic Lateral Sclerosis |
|  | Glutamate Mediated Excitotoxicity in Amyotrophic Lateral Sclerosis |
|  | Microglia and Motor Neuron Interaction Dysregulation |
|  | MIR155 Role in Th17-Cell in Multiple Sclerosis |
|  | SPP1 Induced Survival of Myelin Specific T-Cells in Multiple Sclerosis |
|  | Proteins Involved in Breast Cancer Related to WNT Signaling Pathway |
|  | Proteins Involved in Breast Cancer Related to ESR1 Signaling Pathway |
|  | WNT Signaling in Breast Cancer |
|  | Proteins Involved in Breast Cancer Related to ERBB2/VEGFR/Akt Signaling Pathway |
|  | ERBB/VEGFR/Akt Signaling in Breast Cancer |
|  | IGF1R/AKT Signaling in Breast Cancer |
|  | ESR1/ERBB Positive Luminal Breast Cancer |
|  | Proteins with Altered Expression in Breast Cancer |
|  | Proteins Involved in Breast Cancer Related to IGF1R/Akt Signaling Pathway |
|  | Breast Cancer |
|  | ESR1 Signaling in Breast Cancer |
|  | Endometrial Cancer |
|  | Endometrioid Endometrial Cancer |
|  | Proteins Involved in Endometrial Cancer |
|  | Clear Cell Endometrial Cancer and Papillary Serous Endometrial Cancer |
|  | Proteins with Altered Expression in Endometrial Cancer |
|  | Proteins Involved in Endometriosis |
|  | Angiogenesis in Endometriosis |
|  | Coagulation Factors and PAR1/2 Receptors (F2R/F2RL1) in Endometriosis |
|  | Genes with Mutations Associated with Hereditary Breast and/or Ovarian Cancer Syndrome |
|  | Proteins Involved in High-Grade Serous Ovarian Carcinoma |
|  | Endometrioid Ovarian Carcinoma |
|  | Proteins Involved in Endometrioid Ovarian Carcinoma |
|  | Clear Cell Ovarian Carcinoma |
|  | Proteins Involved in Mucinous Ovarian Carcinoma |
|  | High-Grade Serous Ovarian Carcinoma |
|  | Low-Grade Serous Ovarian Carcinoma |
|  | Proteins Involved in Low-Grade Serous Ovarian Carcinoma |
|  | Ovarian Cancer |
|  | Proteins Involved in Clear Cell Ovarian Carcinoma |
|  | Mucinous Ovarian Carcinoma |
|  | Proteins with Altered Expression in Ovarian Cancer |
|  | Proteins Involved in Primary Ovarian Insufficiency |
|  | LH and FSH Signaling Impairment in Primary Ovarian Insufficiency |
|  | FOXO3 and PTEN Inactivation in Premature Ovarian Failure (Mouse Model) |
|  | MIF Role in Cholesteatoma |
|  | EGF/cyclinD1 Signaling in Acquired Middle Ear Cholesteatoma |
|  | HMGB1/RAGE Signaling in Middle Ear Cholesteatoma |
|  | Cholesteatoma |
|  | HPV E5 Regulates EDNRA and EGFR Signaling Pathway in Keratinocytes |
|  | Androgen Receptor/Akt Signaling |
|  | Fatty Acid Synthase (FASN) Signaling |
|  | Prostate Cancer |
|  | Androgen Receptor/Protein Kinase C-delta Signaling |
|  | Proteins Involved in Prostate Cancer |
|  | EPHA2 Mutations in Cataract |
|  | Steroids Induced Cataract |
|  | VEGFA in Neovascular Glaucoma |
|  | Glutamate Mediated Excitotoxicity in Glaucoma |
|  | Neurotrophic Factor Deprivation in Retinal Ganglion Cell Death |
|  | Proteins Involved in Congenital Deafness |
|  | Immune System Role in Otitis Media |
|  | Alveolar Macrophages Dysfunction in COPD |
|  | Proteins Involved in Chronic Obstructive Pulmonary Disease |
|  | T-Cell Cytotoxic Activation |
|  | Alveolar Epithelial Cell Dysfunction in COPD |
|  | Polycystins Mutation Effects in Cystic Kidney Disease |
|  | Penile Erection Impairment |
|  | Advanced Glycation End Products in Diabetes or Age Related Erectile Dysfunction |
|  | Plasmin Role in Glomerulonephritis |
|  | Endothelial Cell Dysfunction in Glomerulonephritis |
|  | Proteins with Altered Expression in Prostate Cancer |
|  | Ammonia Effects on Brain Cells |
|  | CD157/ITGB2 Signaling in Myeloid Cell |
|  | CD38/CD3 -> JUN/FOS/NF-kB Signaling in T-cell Proliferation |
|  | CD38/CD19 -> JUN/FOS/NF-kB Signaling in B-cell Proliferation |
|  | S100P Expression Target |
|  | HMGB1 Expression Targets |
|  | TTR Expression Targets |
|  | S100B Expression Targets |
|  | AREG -> HIF1A Expression Targets |
|  | AREG -> FOXO3A Expression Target |
|  | Noradrenaline/Gs Expression Targets |
|  | Epinephrine/Gs Expression Targets |
|  | Dopamine/Gi Expression Targets |
|  | Aryl Hydrocarbon Receptor Genomic and non-Genomic Signaling |
|  | AGTR1 -> ARRB1/ARRB2 Signaling |
|  | INSR -> ARRB2/AKT/SRC Signaling |
|  | Hedgehog Family -> ARRB1/ARRB2 Canonical Signaling |
|  | CD19 Expression Targets |
|  | CD81 -> AP-1 Expression Targets |
|  | CD19/CR2 Expression Targets |
|  | CCL3L1 Expression Target |
|  | CCL5 Expression Targets |
|  | CCL7 Expression Target |
|  | IL8 Expression Targets |
|  | Tamoxifen Induced Endometrial Cancer |
|  | Dexamethasone Induced Diabetes |
|  | Dexamethasone Induced Osteoporosis |
|  | Doxorubicin Induced Cardiotoxicity |
|  | Ritonavir Induced Diabetes |
|  | Clozapine Induced Granulocytopenia |
|  | Cyclosporine Induced Nephrotoxicity |
|  | EGF -> HIF1A Expression Targets |
|  | EGF -> FOXO3A Expression Targets |
|  | EGFR/ERBB2 -> HIF1A Signaling |
|  | EREG -> FOXO3A Expression Target |
|  | EREG -> HIF1A Expression Target |
|  | Erythropoietin -> FOXO3A Expression Targets |
|  | Erythropoietin -> NF-kB Expression Targets |
|  | PECAM1 Expression Targets |
|  | F2 -> STAT1/NF-kB Expression Targets |
|  | PLG -> STAT1/NF-kB Expression Targets |
|  | FGF2 -> FOXO3A Expression Targets |
|  | FGF7 -> FOXO3A Expression Target |
|  | FGF10 -> FOXO3A Expression Target |
|  | FSHR Expression Targets |
|  | GH1/GHR -> NF-kB/ELK/SRF/MYC Expression Targets |
|  | CSH1/GHR Expression Targets |
|  | HBEGF -> FOXO3A Expression Target |
|  | HBEGF -> HIF1A Expression Targets |
|  | HGF -> FOXO3A Expression Targets |
|  | HGFR -> FOXO3A Signaling |
|  | TAM Receptor Signaling Activation in Cancer |
|  | PI3K/AKT/MTOR Signaling Activation in Cancer |
|  | Hedgehog Signaling Activation in Cancer |
|  | Proteins with Altered Expression in Cancer-Associated Sustaining of Proliferative Signaling |
|  | Genes with Mutations in Cancer-Associated Sustaining of Proliferative Signaling |
|  | G0/G1 Cell Cycle Phase Transition Activation in Cancer |
|  | RAS/RAF/MAPK Signaling Activation in Cancer |
|  | N2 Neutrophils in Tumor-Promoting Inflammation and Tumor Progression |
|  | RAS/RAF/MAPK Signaling Activation by Blocking of Tumor Suppressors |
|  | Hedgehog Signaling Activation by Blocking of Tumor Suppressors |
|  | TGFB Signaling activation by Blocking of Tumor Suppressors |
|  | PI3K/AKT/MTOR Signaling Activation by Blocking of Tumor Suppressors |
|  | Hippo/YAP1 Signaling Deregulation in Cancer |
|  | Proteins with Altered Expression in Cancer-Associated Resisting to Cell Death |
|  | Genes with Mutations in Cancer-Associated Resisting to Cell Death |
|  | Apoptosis Evasion in Cancer: Overview |
|  | Hypoxia-Induced Cell Death Evasion in Cancer |
|  | Apoptosis Block by BCL2 Family Proteins in Cancer |
|  | HIF1A in Vasculogenic Mimicry of Cancer |
|  | VEGFA Dependent Angiogenesis in Cancer |
|  | ANGPT/TEK Maintains Endothelial Cell Quiescence in Cancer |
|  | Inhibition of THBS1 Activates Angiogenesis in Cancer |
|  | FBLN5 Potentiates Migration and Proliferation of Endothelial Cells in Cancer |
|  | HIF1A as Master Regulator of Angiogenesis in Cancer |
|  | VEGF Independent Angiogenesis in Cancer |
|  | Integrins in Cancer Cell Motility, Invasion and Survival |
|  | Proteins with Altered Expression in Cancer Metastases |
|  | Epithelial to Mesenchymal Transition in Cancer: Overview |
|  | BSG (CD147) in Cancer Cells Motility, Invasion and Survival |
|  | MTOR/TORC in Cancer Cell Motility and Invasion |
|  | Hyaluronic Acid, CD44 and HMMR in Cancer Cell Invasion and Survival |
|  | CDH2 Activation Promotes Cancer Cell Migration and Survival |
|  | TGFB Family in Epithelial to Mesenchymal Transition in Cancer |
|  | Invadopodia Formation in Cancer Cells |
|  | Metabolic Reprogramming in Cancer: Overview |
|  | Skeletal Muscle Wasting in Cancer Cachexia |
|  | Metabolic Effects of Oncogenes and Tumor Suppressor in Cancer Cells |
|  | Lipogenesis/Lipolysis Activation in Cancer Cells and Cancer-Associated Adipocytes |
|  | Glycolysis Activation in Cancer (Warburg Effect) |
|  | Genes with Mutations in Cancer Metabolic Reprogramming |
|  | Proteins with Altered Expression in Cancer Metabolic Reprogramming |
|  | Treg Cells Promote Immunosuppression in Cancer Immune Escape |
|  | IDO1 in Cancer Immune Escape |
|  | Effector T-cell Inactivation in Cancer Immune Escape |
|  | EZH2 Methyltransferase Role in Cancer |
|  | DNMT and MBD Families Activation in DNA Methylation in Cancer |
|  | IGF1 -> CEBPA/FOXO1A Expression Targets |
|  | IGF2 -> CEBPA/FOXO1A Expression Targets |
|  | Insulin -> CEBPA/CTNNB/FOXA/FOXO Expression Targets |
|  | Insulin -> ELK/SRF/HIF1A/MYC/SREBF Expression Targets |
|  | CD19 -> NF-kB Signaling |
|  | AGER -> NF-kB Signaling |
|  | ICAM2 -> CTNNB/FOXO/STAT3 Signaling |
|  | InsulinR -> CTNNB/FOXA/FOXO Signaling |
|  | InsulinR -> ELK/SRF/SREBF Signaling |
|  | IGF1R -> CEBPA/FOXO1A Signaling |
|  | FibronectinR -> CTNNB Signaling |
|  | FibronectinR -> NF-kB Signaling |
|  | MacrophageR -> CEBPB -> NF-kB Signaling |
|  | Fibronectin Expression Targets |
|  | CTGF -> FOXO3A Expression Target |
|  | ICAM1 Expression Targets |
|  | Fibrinogen Expression Targets |
|  | IL7R -> FOXO/NF-kB Signaling |
|  | IL15R -> NF-kB/NFATC Signaling |
|  | IL23R -> STAT3/NF-kB Signaling |
|  | IL12R -> NF-kB/NFATC Signaling |
|  | IL7 Expression Targets |
|  | IL15 Expression Targets |
|  | IL12B Expression Targets |
|  | PAF/Gq -> NF-kB Expression Targets |
|  | Elevated Receptors -> Expression Targets in Lymph Node |
|  | CD Markers -> Expression Targets in Lymphoid System and Blood |
|  | NRG1 -> FOXO3A Expression Targets |
|  | NRG1 -> HIF1A Expression Target |
|  | NGF -> FOXO/MYCN/ELK-SRF Expression Targets |
|  | BDNF Expression Targets |
|  | NTF3 Expression Targets |
|  | NOTCH -> NF-kB Signaling |
|  | DLL4 Expression Targets |
|  | ADAM17 Expression Targets |
|  | JAG1 Expression Targets |
|  | DLL1 Expression Targets |
|  | NOTCH Expression Targets |
|  | Adenosine Expression Targets |
|  | PDGFB -> FOXO3A Expression Target |
|  | PDGF -> FOXO3A Expression Targets |
|  | PDGFR -> FOXO3A Signaling |
|  | PRL/GHR -> NF/kB/ELK/SRF/MYC Expression Targets |
|  | PDCD1 -> ATF/CREB/CREBBP Expression Targets |
|  | PDCD1 -> STAT Expression Targets |
|  | GUCYC2 Signaling |
|  | DDR1 -> NF-kB Signaling |
|  | AngiopoietinR -> FOXO Signaling |
|  | GDNF -> HSF1 Signaling |
|  | EphrinB -> MAPK/JUN/FOS Signaling |
|  | NTRK -> FOXO/MYCN Signaling |
|  | CSF1 -> FOXO3A Expression Targets |
|  | Collagen -> NF-kB Expression Targets |
|  | GDNF -> HSF1 Expression Targets |
|  | Lysophosphatidic Acid/LPARs Signaling |
|  | Androgen Receptor non-Genomic Signaling |
|  | FOXO1 Signaling |
|  | MERTK Signaling |
|  | Ras Signaling |
|  | TGF-beta Signaling |
|  | AXL Receptor Tyrosine Kinase Signaling |
|  | PI3K/RAC1 Signaling |
|  | mTOR Signaling |
|  | TNF-alpha/TNFRSF1B Signaling |
|  | KRAS Signaling |
|  | VEGF Signaling |
|  | Estrogens/ESR1 non-Genomic Signaling |
|  | EGFR Signaling |
|  | HRAS Signaling |
|  | Hedgehog Family Signaling |
|  | T-Cell Receptor -> NF-kB Signaling |
|  | TGFA -> FOXO3A Expression Targets |
|  | TGFA -> HIF1A Expression Targets |
|  | TNFSF10 Expression Targets |
|  | CD40LG -> NF-kB/ELK/SRF -> CREB/NFATC Expression Targets |
|  | TNFSF13B Expression Targets |
|  | TNF -> STAT Expression Targets |
|  | TNFRSF5/13B -> NFATC1 Signaling |
|  | Thrombopoietin -> SPI1 Expression Targets |
|  | Thrombopoietin -> FOXO3A Expression Target |
|  | TLR1 -> 2/6 Expression Targets |
|  | TLR1/2/6 -> NF-kB Signaling |
|  | ER Stress (Unfolded Protein Response) |
|  | Glutamate Mediated Neurotoxicity |
|  | Ca2+ Cytosolic Overload |
|  | ROS in Angiotensin Mediated Cardiovascular Remodeling and Hypertrophy |
|  | VEGFC -> CTNNB Expression Target |
|  | VEGFA -> FOXO3A Expression Targets |
|  | VEGFA -> CTNNB/CTNND Expression Targets |
|  | VEGFR -> CTNNB Signaling |
|  | VEGFR -> FOXO3A Signaling |
|  | n |
|  | ace2 |
|  | cypa |
|  | pprarg neg, uknown targets, ps-positive |
|  | Model of PPARG signaling in psoriasis |
|  | Model of PPARG related pathways in psoriasis (short version) |
|  | New Pathway (4) |
|  | H2A-K120, H2A-K119 and H3-K4 |
|  | Epigenetic Modifications of H2A-K120, H2A-K119 and H3-K4 |
|  | 3\_Increased proliferation of sebocytes in acne vulgaris |
|  | 2\_Interleukin-17 and interleukin-22 signaling in psoriasis |
|  | 1\_The onset of atopic dermatitis |
|  | 1\_Osteoclast-mediated bone resorption in postmenopausal women |
|  | 2\_Osteoblast-mediated bone demineralization |
|  | 3\_TGFB signaling provokes endochondral ossification with osteophyte formation |
|  | 1\_Local estrogen production stimulates ectopic endometrium proliferation |
|  | 3\_Enhanced angiogenesis in endometriosis |
|  | 2\_Impaired steroidogenesis in PCOS |
|  | 3\_Role of neurotrophic factor deprivation in retinal ganglion cell death |
|  | 3\_Arterial wall inflammation and calcification in atherosclerosis |
|  | 2\_Oxidized LDL causes endothelial dysfunction in atherosclerosis |
|  | 1\_Endothelial cells stimulate vasoconstriction and thrombosis in pulmonary hypertension |
|  | 2\_Cardiomyocyte hypertrophy |
|  | 4\_Endothelial cells repress vasoconstriction in hypertension |
|  | 1\_Cigarette smoke causes dysfunction and production of inflammatory mediators in alveolar epithelial cells |
|  | 3\_Role of alveolar macrophages in pulmonary tissue destruction in COPD |
|  | 1\_Th2 cell response in asthma |
|  | 2\_2\_Eosinophilia and neutrophilia in asthma: eosinophil apoptosis |
|  | 4\_2\_Airway smooth muscle cells proliferation and airway tissue remodeling |
|  | 2\_Intestine epithelial cells drive immunological responses in ulcerative colitis |
|  | 1\_Triglyceride storage in NAFLD |
|  | 1\_CD4+ T cell and epithelial cell response in celiac disease |
|  | 4\_Tax-induced disruption of the G1/S transition in HTLV-1 infected T-cell |
|  | 2\_HIV - induced depletion of CD4+ T-cells |
|  | 3\_1\_Beta-cell proliferation and compensation: proliferation |
|  | 1\_1\_Insulin synthesis and secretion: insulin processing and storage |
|  | 3\_3\_Beta-cell proliferation and compensation: recovery |
|  | 4\_2\_Insulin resistance: inflammation-related insulin resistance |
|  | 4\_1\_Insulin resistance: insulin signaling |
|  | 4\_3\_Insulin resistance: FFA-related insulin resistance |
|  | 1\_Antigen-presenting cells promote islet dysfunction and immune system activation in T1D |
|  | 1\_Microangiopathy in diabetic neuropathy |
|  | 2\_Neuron and Schwann cell death |
|  | 1\_Glutamate and calcium excitotoxicity of motor neurons in ALS |
|  | 1\_1\_Glutamate overload enforced prostaglandins and NO synthesis in neurons |
|  | 2\_1\_General pathways of thyroid hormone signaling |
|  | drug-target pairs direct regulation |
|  | New Combined Pathway |
|  | Model of PPARG signaling in psoriais (tested) |
|  | DIPG overview\_1 (histones) |
|  | DIPG non histone mutations |
|  | DIPG histone 3 mutations |
|  | New Pathway |
|  | DIPG overview\_2 |
|  | HRAS->RAF->MAPK1/3 |
|  | mapk1 protein modificators |
|  | Interleukin-17 and interleukin-22 signaling in psoriasis |

---

|  |  |
| --- | --- |
| Group | Genes with Mutations Associated with Ageing (non-Mammalian Animal Model) |
|  | Proteins Involved in Cowden Syndrome |
|  | Genes with Mutations Associated with Cowden Syndrome |

---

|  |  |
| --- | --- |
| Source | curated |
|  | ResNet |
|  | GO component |
|  | Biopax |

---

|  |  |
| --- | --- |
| MedScan ID | 207 |

---

|  |  |
| --- | --- |
| LocusLink ID | 207 |
|  | 11651 |
|  | 24185 |
|  | 50586 |
|  | 280991 |
|  | 569086 |
|  | 201 |
|  | 268604 |
|  | 111651 |
|  | 210766 |

---

|  |  |
| --- | --- |
| Alias | Akt kinase |
|  | v-akt murine thymoma viral oncogene homolog 1 |
|  | Akt/PKBs |
|  | RAC protein kinase alpha RAC-PK alpha |
|  | 6.1a protein |
|  | Akt |
|  | related to A and C kinases |
|  | OTTMUSP00000022947 |
|  | thymoma viral proto-oncogene |
|  | rac protein kinase alpha |
|  | MGC29148 |
|  | CWS6 |
|  | C-AKT |
|  | PKB/Akt |
|  | RAC-PK-alpha |
|  | protein kinase Akt |
|  | PKB/Akts |
|  | RAC-alpha serine/threonine kinase |
|  | Akt/PKB |
|  | protein kinase B-alpha |
|  | tumor rejection antigen peptide pRLib |
|  | protein kinase B alpha |
|  | RAC-ALPHA |
|  | thymoma viral proto-oncogene 1; thymoma viral proto-oncogene |
|  | thymoma viral proto-oncogene I |
|  | LOC217879 |
|  | v-akt murine thymoma viral oncogene-like protein 1 |
|  | OTTMUSP00000022944 |
|  | serine/threonine protein kinase B |
|  | LOC268604 |
|  | PKB alpha |
|  | v-akt murine thymoma viral oncogene homologue 1 |
|  | v-akt murine thymoma viral oncogene homolog I |
|  | Murine thymoma viral (v-akt) oncogene homolog I |
|  | PRKBA |
|  | AKT1 kinase |
|  | thymoma viral proto-oncogene 1 |
|  | Akt1 |
|  | Protein kinase B |
|  | murine thymoma viral (v-akt) oncogene homolog 1 |
|  | AKT serine/threonine kinase 1 |
|  | MGC99656 |
|  | OTTMUSP00000022949 |
|  | Akt I |
|  | AKTI |
|  | PKB |
|  | proto-oncogene c-Akt |
|  | LTR-akt |
|  | proto-oncogene proteins c-akt |
|  | protooncogene protein c akt |
|  | AKT1m |
|  | RAC |
|  | PKB-ALPHA |
|  | serine-threonine protein kinase |
|  | RAC-alpha serine/threonine-protein kinase |
|  | PKBalpha |
|  | Akt1m protein |
|  | C6.1A |
|  | murine thymoma viral (v-akt) oncogene homolog-1 |
|  | KRAC |
|  | serine/threonine protein kinase |
|  | KRAC\_HUMAN |
|  | Akt1/PKB protein kinase |
|  | Protein kinase B |
|  | amelogenesis imperfecta 3, hypomaturation or hypoplastic type |
|  | AIH3 |
|  | similar to thymoma viral proto-oncogene 1; thymoma viral proto-oncogene |
|  | LOC569086 |
|  | chromosome |
|  | scaffold |
|  | similar to protein kinase N2 |
|  | Ms6-1 |
|  | minisatellite I |
|  | minisatellite-1 detected by probe 33 |
|  | AKT1 protein |
|  | minisatellite-1 |
|  | minisatellite-1 detected by probe 33.6 |
|  | Ms6 I |

---

|  |  |
| --- | --- |
| EC Number | 2.7.11.1 |
|  | 2.7.1.37 |
|  | 2.7.1.- |

---

|  |  |
| --- | --- |
| GO ID | 0071889 |
|  | 0005524 |
|  | 0032794 |
|  | 0005516 |
|  | 0019899 |
|  | 0016301 |
|  | 0030235 |
|  | 0005547 |
|  | 0043325 |
|  | 0042803 |
|  | 0005080 |
|  | 0004672 |
|  | 0051721 |
|  | 0004674 |
|  | 0004712 |
|  | 0007186 |
|  | 0007249 |
|  | 0038061 |
|  | 0031295 |
|  | 0031929 |
|  | 0032148 |
|  | 0006924 |
|  | 0007568 |
|  | 0043276 |
|  | 0008637 |
|  | 0008643 |
|  | 0030154 |
|  | 0002042 |
|  | 0030030 |
|  | 0008283 |
|  | 0006464 |
|  | 0006974 |
|  | 0071276 |
|  | 0071364 |
|  | 0097011 |
|  | 0071456 |
|  | 0032869 |
|  | 0071260 |
|  | 1990090 |
|  | 0071407 |
|  | 0140052 |
|  | 0071380 |
|  | 0034614 |
|  | 0071356 |
|  | 0035924 |
|  | 0051186 |
|  | 0019221 |
|  | 0031018 |
|  | 0007173 |
|  | 0072655 |
|  | 0060079 |
|  | 0097194 |
|  | 0007281 |
|  | 0042593 |
|  | 0006006 |
|  | 0005978 |
|  | 0060709 |
|  | 0030212 |
|  | 0006954 |
|  | 0008286 |
|  | 0048009 |
|  | 0035655 |
|  | 0035556 |
|  | 0060716 |
|  | 0031663 |
|  | 0072656 |
|  | 0060644 |
|  | 0001893 |
|  | 0046329 |
|  | 0045746 |
|  | 0043066 |
|  | 0010507 |
|  | 0045792 |
|  | 0043154 |
|  | 0010951 |
|  | 2001240 |
|  | 0031999 |
|  | 0010629 |
|  | 1903038 |
|  | 2000402 |
|  | 0016242 |
|  | 1901215 |
|  | 1902176 |
|  | 0010748 |
|  | 0032091 |
|  | 0051898 |
|  | 0006469 |
|  | 0100002 |
|  | 0071901 |
|  | 0031397 |
|  | 0045861 |
|  | 0090201 |
|  | 0006809 |
|  | 0001649 |
|  | 0018105 |
|  | 0018107 |
|  | 0032287 |
|  | 0014065 |
|  | 0016310 |
|  | 0051091 |
|  | 1900087 |
|  | 1903721 |
|  | 0043065 |
|  | 0043536 |
|  | 0030307 |
|  | 0008284 |
|  | 0032270 |
|  | 0045737 |
|  | 0032079 |
|  | 0001938 |
|  | 0045742 |
|  | 0045600 |
|  | 0010763 |
|  | 0010628 |
|  | 0046326 |
|  | 0010907 |
|  | 0045725 |
|  | 0046889 |
|  | 0010918 |
|  | 0045429 |
|  | 0051000 |
|  | 0046622 |
|  | 0033138 |
|  | 0032436 |
|  | 2000010 |
|  | 1900182 |
|  | 1903078 |
|  | 0001934 |
|  | 0048661 |
|  | 0010765 |
|  | 0045944 |
|  | 0045893 |
|  | 0045907 |
|  | 0046777 |
|  | 0030163 |
|  | 0006606 |
|  | 0043491 |
|  | 0006468 |
|  | 0016567 |
|  | 0042981 |
|  | 0030334 |
|  | 0005979 |
|  | 0043488 |
|  | 0031641 |
|  | 0010975 |
|  | 0050999 |
|  | 1901796 |
|  | 0006417 |
|  | 0070141 |
|  | 0034405 |
|  | 0032094 |
|  | 0060416 |
|  | 0009408 |
|  | 1990418 |
|  | 0006979 |
|  | 0007165 |
|  | 0021510 |
|  | 0051146 |
|  | 0006412 |
|  | 0005911 |
|  | 0036064 |
|  | 0005737 |
|  | 0005829 |
|  | 0015630 |
|  | 0005739 |
|  | 0005654 |
|  | 0005634 |
|  | 0005886 |
|  | 0098794 |
|  | 0032991 |
|  | 0005819 |
|  | 0031982 |
|  | 0000166 |
|  | 0019901 |
|  | 0016740 |
|  | 0006915 |
|  | 0005975 |
|  | 0036294 |
|  | 0071363 |
|  | 1901653 |
|  | 0007010 |
|  | 0005977 |
|  | 0007275 |
|  | 0007399 |
|  | 0032880 |
|  | 0009725 |
|  | 0010033 |
|  | 0016020 |
|  | 2001047 |
|  | 1901976 |
|  | 0044281 |
|  | 0014066 |
|  | 0038128 |
|  | 0005543 |
|  | 0010467 |
|  | 0099565 |
|  | 0030168 |
|  | 0016772 |
|  | 0042802 |
|  | 0005622 |
|  | 0030027 |
|  | 0045087 |
|  | 0045768 |
|  | 0045884 |
|  | 0016071 |
|  | 0016070 |
|  | 0000060 |
|  | 0005515 |
|  | 0038095 |
|  | 0043234 |
|  | 0006810 |
|  | 0048010 |
|  | 0048011 |
|  | 0048015 |
|  | 0007596 |
|  | 0097193 |
|  | 0006916 |
|  | 0015758 |
|  | 0008543 |
|  | 0031659 |
|  | 1900740 |
|  | 0008633 |
|  | 0090004 |
|  | 0046209 |
|  | 0061024 |
|  | 0042640 |
|  | 0008629 |
|  | 0030155 |
|  | 0005625 |
|  | 0004713 |
|  | 0005351 |
|  | 0001568 |
|  | 0007242 |
|  | 0001890 |
|  | 0040018 |
|  | 0070201 |
|  | 0005856 |
|  | 0006445 |
|  | 0000074 |
|  | 0050875 |
|  | 0004702 |
|  | 0008151 |
|  | 0007048 |
|  | 0023034 |
|  | 0001764 |
|  | 0010165 |
|  | 0000151 |
|  | 0030234 |
|  | 2001243 |

---

|  |  |
| --- | --- |
| KEGG ID | hsa:207 |
|  | mmu:11651 |
|  | rno:24185 |

---

|  |  |
| --- | --- |
| MedLine Reference | 10400692 |
|  | 11572795 |
|  | 11809761 |
|  | 11834296 |
|  | 11904450 |
|  | 12084817 |
|  | 12089143 |
|  | 12095990 |
|  | 12112022 |
|  | 12140289 |
|  | 12145204 |
|  | 12194869 |
|  | 12198247 |
|  | 12392981 |
|  | 12496370 |
|  | 12543452 |
|  | 12586553 |
|  | 12624428 |
|  | 12734207 |
|  | 12761242 |
|  | 12767043 |
|  | 12789537 |
|  | 12810818 |
|  | 12816542 |
|  | 12816756 |
|  | 12818177 |
|  | 12829832 |
|  | 12837666 |
|  | 12900409 |
|  | 13129932 |
|  | 14517300 |
|  | 14522978 |
|  | 14523027 |
|  | 14565960 |
|  | 14576824 |
|  | 14581516 |
|  | 14596918 |
|  | 14607273 |
|  | 14670845 |
|  | 14698990 |
|  | 14707121 |
|  | 14753441 |
|  | 15007074 |
|  | 15024023 |
|  | 15060019 |
|  | 15065872 |
|  | 15120593 |
|  | 15149850 |
|  | 15203192 |
|  | 15282275 |
|  | 15289442 |
|  | 15319368 |
|  | 15337841 |
|  | 15375154 |
|  | 15451022 |
|  | 15466647 |
|  | 15489316 |
|  | 15496504 |
|  | 15501455 |
|  | 15511630 |
|  | 15574336 |
|  | 15590660 |
|  | 15616009 |
|  | 15632127 |
|  | 15637121 |
|  | 15694367 |
|  | 15706595 |
|  | 15780591 |
|  | 15797868 |
|  | 15809304 |
|  | 15814837 |
|  | 15845076 |
|  | 15848222 |
|  | 15853465 |
|  | 15856020 |
|  | 15890358 |
|  | 15890643 |
|  | 15896702 |
|  | 15896972 |
|  | 15979279 |
|  | 16027121 |
|  | 16027165 |
|  | 16046294 |
|  | 16049005 |
|  | 16061478 |
|  | 16087293 |
|  | 16098714 |
|  | 16123202 |
|  | 16171808 |
|  | 16181409 |
|  | 16237183 |
|  | 16269516 |
|  | 16282323 |
|  | 16316997 |
|  | 16337154 |
|  | 16343426 |
|  | 16428351 |
|  | 16440211 |
|  | 16452480 |
|  | 16466740 |
|  | 16531507 |
|  | 16532026 |
|  | 16540465 |
|  | 16567807 |
|  | 16596723 |
|  | 16642037 |
|  | 16679308 |
|  | 16717100 |
|  | 16719794 |
|  | 16794215 |
|  | 16843447 |
|  | 16855220 |
|  | 16864652 |
|  | 16949134 |
|  | 16956580 |
|  | 16966378 |
|  | 16978869 |
|  | 17109063 |
|  | 17142780 |
|  | 7999118 |
|  | 9632753 |
|  | 10349636 |
|  | 10428027 |
|  | 10485701 |
|  | 10521450 |
|  | 10579998 |
|  | 10802656 |
|  | 10811865 |
|  | 10922068 |
|  | 11042159 |
|  | 11076861 |
|  | 11086988 |
|  | 11217851 |
|  | 11238953 |
|  | 11282895 |
|  | 11376107 |
|  | 11533044 |
|  | 11544177 |
|  | 11549290 |
|  | 11590437 |
|  | 11731234 |
|  | 11752635 |
|  | 11777951 |
|  | 11861503 |
|  | 11909972 |
|  | 11943770 |
|  | 11950275 |
|  | 11956204 |
|  | 11997518 |
|  | 12015964 |
|  | 12031847 |
|  | 12032842 |
|  | 12042314 |
|  | 12052887 |
|  | 12084724 |
|  | 12091320 |
|  | 12097372 |
|  | 12124386 |
|  | 12130510 |
|  | 12134068 |
|  | 12140361 |
|  | 12146978 |
|  | 12150915 |
|  | 12162464 |
|  | 12163490 |
|  | 12217896 |
|  | 12237475 |
|  | 12244155 |
|  | 12270033 |
|  | 12388598 |
|  | 12419217 |
|  | 12446787 |
|  | 12466851 |
|  | 12477932 |
|  | 12517744 |
|  | 12524439 |
|  | 12525499 |
|  | 12529330 |
|  | 12531698 |
|  | 12574380 |
|  | 12592384 |
|  | 12670507 |
|  | 12671688 |
|  | 12705474 |
|  | 12717433 |
|  | 12718435 |
|  | 12761849 |
|  | 12766772 |
|  | 12773302 |
|  | 12773577 |
|  | 12776124 |
|  | 12782295 |
|  | 12782654 |
|  | 12783884 |
|  | 12791994 |
|  | 12807428 |
|  | 12808085 |
|  | 12837294 |
|  | 12851395 |
|  | 12865424 |
|  | 12869584 |
|  | 12884297 |
|  | 12890675 |
|  | 12904304 |
|  | 12938156 |
|  | 12944477 |
|  | 12944902 |
|  | 14502571 |
|  | 14522959 |
|  | 14522993 |
|  | 14559920 |
|  | 14560023 |
|  | 14561707 |
|  | 14595115 |
|  | 14597666 |
|  | 14610089 |
|  | 14623889 |
|  | 14633850 |
|  | 14656892 |
|  | 14657000 |
|  | 14691534 |
|  | 14701865 |
|  | 14730361 |
|  | 14744754 |
|  | 14744864 |
|  | 14747529 |
|  | 14749368 |
|  | 14749824 |
|  | 14755341 |
|  | 14966566 |
|  | 14967838 |
|  | 14985363 |
|  | 14985505 |
|  | 14988230 |
|  | 15010863 |
|  | 15020298 |
|  | 15046607 |
|  | 15081586 |
|  | 15100285 |
|  | 15100319 |
|  | 15105289 |
|  | 15107468 |
|  | 15121188 |
|  | 15128676 |
|  | 15166036 |
|  | 15169915 |
|  | 15192111 |
|  | 15192701 |
|  | 15199124 |
|  | 15207235 |
|  | 15217935 |
|  | 15226309 |
|  | 15241468 |
|  | 15247222 |
|  | 15254091 |
|  | 15278907 |
|  | 15280422 |
|  | 15309026 |
|  | 15322182 |
|  | 15345655 |
|  | 15351728 |
|  | 15361852 |
|  | 15364809 |
|  | 15381256 |
|  | 15389876 |
|  | 15448167 |
|  | 15456827 |
|  | 15459205 |
|  | 15467831 |
|  | 15485887 |
|  | 15489897 |
|  | 15509711 |
|  | 15527798 |
|  | 15528226 |
|  | 15537637 |
|  | 15546863 |
|  | 15572359 |
|  | 15572689 |
|  | 15578100 |
|  | 15579468 |
|  | 15580267 |
|  | 15582777 |
|  | 15583002 |
|  | 15611473 |
|  | 15663476 |
|  | 15678105 |
|  | 15695173 |
|  | 15705789 |
|  | 15713628 |
|  | 15713641 |
|  | 15723049 |
|  | 15753085 |
|  | 15757972 |
|  | 15761494 |
|  | 15764603 |
|  | 15769848 |
|  | 15775979 |
|  | 15782199 |
|  | 15797869 |
|  | 15799971 |
|  | 15817464 |
|  | 15833736 |
|  | 15836427 |
|  | 15840586 |
|  | 15842198 |
|  | 15887120 |
|  | 15946254 |
|  | 15967113 |
|  | 15994435 |
|  | 16000198 |
|  | 16005454 |
|  | 16007268 |
|  | 16036916 |
|  | 16036919 |
|  | 16049178 |
|  | 16075056 |
|  | 16083985 |
|  | 16116448 |
|  | 16135819 |
|  | 16141072 |
|  | 16141073 |
|  | 16141217 |
|  | 16151855 |
|  | 16169459 |
|  | 16179343 |
|  | 16203862 |
|  | 16204028 |
|  | 16207820 |
|  | 16227992 |
|  | 16239343 |
|  | 16263172 |
|  | 16287854 |
|  | 16293788 |
|  | 16298682 |
|  | 16310342 |
|  | 16338928 |
|  | 16352665 |
|  | 16354698 |
|  | 16357133 |
|  | 16375864 |
|  | 16453020 |
|  | 16453021 |
|  | 16478789 |
|  | 16478996 |
|  | 16484540 |
|  | 16487036 |
|  | 16495456 |
|  | 16510873 |
|  | 16511503 |
|  | 16513830 |
|  | 16516160 |
|  | 16537363 |
|  | 16581799 |
|  | 16613850 |
|  | 16613861 |
|  | 16626396 |
|  | 16645141 |
|  | 16680151 |
|  | 16682621 |
|  | 16687609 |
|  | 16704410 |
|  | 16707461 |
|  | 16741926 |
|  | 16763066 |
|  | 16778075 |
|  | 16787946 |
|  | 16797529 |
|  | 16798838 |
|  | 16840722 |
|  | 16880210 |
|  | 16882732 |
|  | 16882883 |
|  | 16886062 |
|  | 16889755 |
|  | 16904613 |
|  | 16914513 |
|  | 16916926 |
|  | 16917099 |
|  | 16923802 |
|  | 16923958 |
|  | 16950125 |
|  | 16950770 |
|  | 16963136 |
|  | 16966610 |
|  | 16973750 |
|  | 16982699 |
|  | 17012749 |
|  | 17015449 |
|  | 17015619 |
|  | 17019809 |
|  | 17043309 |
|  | 17045205 |
|  | 17052453 |
|  | 17068339 |
|  | 17077150 |
|  | 17124499 |
|  | 17141631 |
|  | 17202487 |
|  | 1794043 |
|  | 7585504 |
|  | 8437858 |
|  | 8889548 |
|  | 9415393 |
|  | 9620559 |
|  | 10082674 |
|  | 10085094 |
|  | 10102273 |
|  | 10217147 |
|  | 10224060 |
|  | 10226025 |
|  | 10358014 |
|  | 10376602 |
|  | 10376603 |
|  | 10438924 |
|  | 10454575 |
|  | 10485710 |
|  | 10485711 |
|  | 10490823 |
|  | 10491192 |
|  | 10497255 |
|  | 10527852 |
|  | 10542266 |
|  | 10570282 |
|  | 10576742 |
|  | 10617634 |
|  | 10635328 |
|  | 10698680 |
|  | 10748004 |
|  | 10833263 |
|  | 10866658 |
|  | 10869359 |
|  | 10872470 |
|  | 10896934 |
|  | 10910062 |
|  | 10926925 |
|  | 10930578 |
|  | 10958679 |
|  | 10983986 |
|  | 10995457 |
|  | 11035810 |
|  | 11042204 |
|  | 11116148 |
|  | 11139588 |
|  | 11154276 |
|  | 11154281 |
|  | 11156376 |
|  | 11156964 |
|  | 11274386 |
|  | 11278835 |
|  | 11313479 |
|  | 11404460 |
|  | 11410590 |
|  | 11410591 |
|  | 11438723 |
|  | 11445557 |
|  | 11504915 |
|  | 11535620 |
|  | 11546794 |
|  | 11579209 |
|  | 11583630 |
|  | 11585925 |
|  | 11598301 |
|  | 11689711 |
|  | 11696579 |
|  | 11707444 |
|  | 11707464 |
|  | 11710897 |
|  | 11756412 |
|  | 11777913 |
|  | 11782427 |
|  | 11809791 |
|  | 11825911 |
|  | 11828257 |
|  | 11855836 |
|  | 11877387 |
|  | 11884598 |
|  | 11902142 |
|  | 11903042 |
|  | 11923280 |
|  | 11956222 |
|  | 11960368 |
|  | 11976320 |
|  | 11994271 |
|  | 11994280 |
|  | 12009899 |
|  | 12011046 |
|  | 12062056 |
|  | 12062094 |
|  | 12070137 |
|  | 12077256 |
|  | 12138087 |
|  | 12138205 |
|  | 12149249 |
|  | 12167664 |
|  | 12167717 |
|  | 12176337 |
|  | 12176338 |
|  | 12176997 |
|  | 12218048 |
|  | 12244301 |
|  | 12244302 |
|  | 12244303 |
|  | 12297295 |
|  | 12376533 |
|  | 12393747 |
|  | 12438239 |
|  | 12446767 |
|  | 12458207 |
|  | 12527373 |
|  | 12529294 |
|  | 12535517 |
|  | 12592338 |
|  | 12621049 |
|  | 12637511 |
|  | 12687010 |
|  | 12722480 |
|  | 12727836 |
|  | 12734380 |
|  | 12741986 |
|  | 12757707 |
|  | 12768030 |
|  | 12783873 |
|  | 12808093 |
|  | 12853467 |
|  | 12874217 |
|  | 12888921 |
|  | 12919677 |
|  | 12952968 |
|  | 12972603 |
|  | 14500673 |
|  | 14520710 |
|  | 14523021 |
|  | 14534536 |
|  | 14566704 |
|  | 14568991 |
|  | 14611643 |
|  | 14612949 |
|  | 14625285 |
|  | 14636889 |
|  | 14645242 |
|  | 14647146 |
|  | 14679206 |
|  | 14702039 |
|  | 14713953 |
|  | 14742298 |
|  | 14749367 |
|  | 14755253 |
|  | 14973226 |
|  | 14985374 |
|  | 15001646 |
|  | 15010337 |
|  | 15016818 |
|  | 15026145 |
|  | 15047604 |
|  | 15047866 |
|  | 15060171 |
|  | 15067058 |
|  | 15102693 |
|  | 15115729 |
|  | 15136501 |
|  | 15146197 |
|  | 15156201 |
|  | 15187088 |
|  | 15187138 |
|  | 15194442 |
|  | 15203190 |
|  | 15242771 |
|  | 15262962 |
|  | 15262978 |
|  | 15263908 |
|  | 15271644 |
|  | 15283964 |
|  | 15284024 |
|  | 15316080 |
|  | 15326564 |
|  | 15331441 |
|  | 1533586 |
|  | 15342781 |
|  | 15385469 |
|  | 15388791 |
|  | 15389633 |
|  | 15465021 |
|  | 15467757 |
|  | 15475446 |
|  | 15522255 |
|  | 15525681 |
|  | 15533996 |
|  | 15539407 |
|  | 15545271 |
|  | 15545519 |
|  | 15574337 |
|  | 15581622 |
|  | 15607733 |
|  | 15609321 |
|  | 15665327 |
|  | 15665818 |
|  | 15668028 |
|  | 15710591 |
|  | 15718470 |
|  | 15722337 |
|  | 15743789 |
|  | 15778376 |
|  | 15784622 |
|  | 15802268 |
|  | 15806160 |
|  | 15817639 |
|  | 15831676 |
|  | 15837948 |
|  | 15845651 |
|  | 15861136 |
|  | 15879168 |
|  | 15888491 |
|  | 15896313 |
|  | 15905178 |
|  | 15911620 |
|  | 15922745 |
|  | 15961723 |
|  | 15987444 |
|  | 15993382 |
|  | 15994200 |
|  | 15998799 |
|  | 16007163 |
|  | 16007172 |
|  | 16007182 |
|  | 16012720 |
|  | 16026766 |
|  | 16027169 |
|  | 16049004 |
|  | 16049961 |
|  | 16055703 |
|  | 16091017 |
|  | 16099944 |
|  | 16114056 |
|  | 16115861 |
|  | 16128748 |
|  | 16139227 |
|  | 16142341 |
|  | 16148125 |
|  | 16160160 |
|  | 16163388 |
|  | 16174774 |
|  | 16177823 |
|  | 16202996 |
|  | 16216590 |
|  | 16224021 |
|  | 16243840 |
|  | 16263580 |
|  | 16274701 |
|  | 16278380 |
|  | 16280327 |
|  | 16293694 |
|  | 16338927 |
|  | 16365168 |
|  | 16373405 |
|  | 16405925 |
|  | 16407258 |
|  | 16407271 |
|  | 16412424 |
|  | 16428439 |
|  | 16434970 |
|  | 16466644 |
|  | 16474850 |
|  | 16478297 |
|  | 16508008 |
|  | 16532036 |
|  | 16537497 |
|  | 16543232 |
|  | 16549426 |
|  | 16551632 |
|  | 16564100 |
|  | 16568092 |
|  | 16571745 |
|  | 16583435 |
|  | 16601838 |
|  | 16616456 |
|  | 16644866 |
|  | 16651639 |
|  | 16670089 |
|  | 16682946 |
|  | 16684529 |
|  | 16723503 |
|  | 16732486 |
|  | 16763222 |
|  | 16774943 |
|  | 16775835 |
|  | 16785234 |
|  | 16790501 |
|  | 16790529 |
|  | 16820098 |
|  | 16822839 |
|  | 16839745 |
|  | 16840713 |
|  | 16844778 |
|  | 16849522 |
|  | 16897758 |
|  | 16926151 |
|  | 16932738 |
|  | 16962653 |
|  | 16972255 |
|  | 16977260 |
|  | 16981720 |
|  | 16987028 |
|  | 16987250 |
|  | 16988010 |
|  | 16990258 |
|  | 17008323 |
|  | 17015676 |
|  | 17041888 |
|  | 17060455 |
|  | 17141917 |
|  | 1718748 |
|  | 1851997 |
|  | 3037531 |
|  | 3384441 |
|  | 7774014 |
|  | 7891724 |
|  | 8524413 |
|  | 8978681 |
|  | 9005852 |
|  | 9019819 |
|  | 9368760 |
|  | 9381178 |
|  | 9394803 |
|  | 9395488 |
|  | 9736715 |
|  | 9812896 |
|  | 9829964 |
|  | 9973250 |
|  | 11508278 |
|  | 14761976 |
|  | 15118108 |
|  | 15489334 |
|  | 16820947 |
|  | 16842970 |
|  | 16862180 |
|  | 16916907 |
|  | 16946008 |
|  | 17006756 |
|  | 17016437 |
|  | 17024099 |
|  | 17043653 |
|  | 17050554 |
|  | 17065200 |
|  | 17081983 |
|  | 17088265 |
|  | 17097759 |
|  | 17098228 |
|  | 17099727 |
|  | 17113264 |
|  | 17128418 |
|  | 17136479 |
|  | 17136481 |
|  | 17143278 |
|  | 17148458 |
|  | 17151107 |
|  | 17169805 |
|  | 17173971 |
|  | 17179152 |
|  | 17214972 |
|  | 17215852 |
|  | 17235455 |
|  | 17244606 |
|  | 17276404 |
|  | 17287851 |
|  | 17299206 |
|  | 17307335 |
|  | 17310852 |
|  | 17317726 |
|  | 17317825 |
|  | 17318262 |
|  | 17333095 |
|  | 17335965 |
|  | 17342093 |
|  | 17351066 |
|  | 17375124 |
|  | 17383860 |
|  | 17386267 |
|  | 17400190 |
|  | 17404186 |
|  | 17428466 |
|  | 17443665 |
|  | 17458890 |
|  | 17461449 |
|  | 17462862 |
|  | 17469127 |
|  | 17470458 |
|  | 17478078 |
|  | 17483438 |
|  | 17493174 |
|  | 17495954 |
|  | 17495959 |
|  | 17504381 |
|  | 17507655 |
|  | 17507800 |
|  | 17513297 |
|  | 17513702 |
|  | 17520698 |
|  | 17522055 |
|  | 17544405 |
|  | 17544543 |
|  | 17549359 |
|  | 17553806 |
|  | 17565979 |
|  | 17569669 |
|  | 17584963 |
|  | 17606718 |
|  | 17611497 |
|  | 17612563 |
|  | 17616681 |
|  | 17616684 |
|  | 17616691 |
|  | 17638918 |
|  | 17725107 |
|  | 9512493 |
|  | 15249583 |
|  | 15678128 |
|  | 16286931 |
|  | 16514068 |
|  | 16537919 |
|  | 16636172 |
|  | 16889766 |
|  | 16962829 |
|  | 17021050 |
|  | 17081778 |
|  | 17082189 |
|  | 17181553 |
|  | 17209049 |
|  | 17215071 |
|  | 17220321 |
|  | 17251350 |
|  | 17287359 |
|  | 17290308 |
|  | 17311300 |
|  | 17322282 |
|  | 17322895 |
|  | 17332061 |
|  | 17332510 |
|  | 17369192 |
|  | 17382199 |
|  | 17394208 |
|  | 17396120 |
|  | 17397400 |
|  | 17407381 |
|  | 17412896 |
|  | 17417601 |
|  | 17428826 |
|  | 17434462 |
|  | 17439945 |
|  | 17446044 |
|  | 17460049 |
|  | 17464994 |
|  | 17485440 |
|  | 17486091 |
|  | 17490611 |
|  | 17517888 |
|  | 17521884 |
|  | 17531194 |
|  | 17537803 |
|  | 17562714 |
|  | 17609365 |
|  | 16820027 |
|  | 16944316 |
|  | 17016676 |
|  | 17203219 |
|  | 17272355 |
|  | 17272774 |
|  | 17317782 |
|  | 17371807 |
|  | 17496209 |
|  | 17545147 |
|  | 17555943 |
|  | 17628541 |
|  | 17629454 |
|  | 17632284 |
|  | 12244133 |
|  | 14588144 |
|  | 15501927 |
|  | 15808505 |
|  | 16172287 |
|  | 16816403 |
|  | 16963022 |

---

|  |  |
| --- | --- |
| Organism | Homo sapiens {Organism urn:agi-taxid:9606} |
|  | Mus musculus {Organism urn:agi-taxid:10090} |
|  | Rattus norvegicus {Organism urn:agi-taxid:10116} |
|  | Homo sapiens |
|  | Mus musculus |
|  | Rattus norvegicus |
|  | Bos taurus |
|  | Danio rerio |
|  | Human |

---

|  |  |
| --- | --- |
| Mouse chromosome position | 12 61.2 cM |
|  | 12 57.0 cM |

---

|  |  |
| --- | --- |
| OMIM ID | 164730 |
|  | 114500 |
|  | 615109 |
|  | 114480 |
|  | 167000 |
|  | 176920 |
|  | 181500 |
|  | 604370 |
|  | OMIM:164730 |
|  | OMIM:181500 |
|  | OMIM:301201 |

---

|  |  |
| --- | --- |
| Rat chromosome position | 6q32 |

---

|  |  |
| --- | --- |
| Hugo ID | 391 |
|  | HGNC:391 |

---

|  |  |
| --- | --- |
| Human chromosome position | 14q32.33 |
|  | 14q32.32 |

---

|  |  |
| --- | --- |
| Swiss-Prot Accession | B0LPE5 |
|  | P31749 |
|  | B3KVH4 |
|  | P31749.2 |
|  | P31750 |
|  | P31750.2 |
|  | P47196 |
|  | P47196.1 |
|  | B2RAM5 |
|  | B7Z5R1 |
|  | Q9BWB6 |
|  | Q62274 |
|  | Q6GSA6 |
|  | Q9BV07 |
|  | Q8BS26 |
|  | Q6GSA6\_MOUSE |
|  | AKT1\_HUMAN |
|  | AKT1\_RAT |
|  | B0LPE5\_HUMAN |
|  | B3KVH4\_HUMAN |
|  | Q6ZMW5 |

---

|  |  |
| --- | --- |
| PIR ID | A39360 |
|  | S33364 |
|  | JC2437 |

---

|  |  |
| --- | --- |
| GenBank ID | NC\_000014 |
|  | XR\_002957536 |
|  | NM\_001014431 |
|  | NP\_001014431 |
|  | NM\_001014432 |
|  | NP\_001014432 |
|  | NM\_005163 |
|  | NP\_005154 |
|  | NG\_012188 |
|  | A62733 |
|  | CAA03677 |
|  | A63232 |
|  | CAA03712 |
|  | AH011307 |
|  | AL583722 |
|  | AL590327 |
|  | CH471061 |
|  | EAW81881 |
|  | EAW81882 |
|  | EAW81883 |
|  | EU332835 |
|  | ABY87524 |
|  | JC006886 |
|  | CDL93491 |
|  | JC604553 |
|  | CEF39425 |
|  | JC604555 |
|  | CEF39426 |
|  | JC604557 |
|  | CEF39427 |
|  | JC739425 |
|  | CEF39431 |
|  | JX512440 |
|  | AGC09587 |
|  | KT583844 |
|  | KT585272 |
|  | KU639689 |
|  | AMR60723 |
|  | KU639690 |
|  | AMR60724 |
|  | KU639691 |
|  | AMR60725 |
|  | KU639692 |
|  | AMR60726 |
|  | KU639693 |
|  | AMR60727 |
|  | KU639694 |
|  | AMR60728 |
|  | KU639695 |
|  | AMR60729 |
|  | KU639696 |
|  | AMR60730 |
|  | KU639697 |
|  | AMR60731 |
|  | KU639698 |
|  | AMR60732 |
|  | KU639699 |
|  | AMR60733 |
|  | KU639700 |
|  | AMR60734 |
|  | KU639701 |
|  | AMR60735 |
|  | KU639702 |
|  | AMR60736 |
|  | KU639703 |
|  | AMR60737 |
|  | KU639704 |
|  | AMR60738 |
|  | KU639705 |
|  | AMR60739 |
|  | LN845722 |
|  | LN845723 |
|  | LN845724 |
|  | LN845725 |
|  | LN845726 |
|  | LN845727 |
|  | LN845728 |
|  | LN845729 |
|  | LN845730 |
|  | LN845731 |
|  | LN845732 |
|  | LN845733 |
|  | LN845734 |
|  | LN845735 |
|  | LN845736 |
|  | LN845737 |
|  | LN845738 |
|  | LN845739 |
|  | LN845740 |
|  | LN845741 |
|  | LN845742 |
|  | LN845743 |
|  | LN845744 |
|  | LN845745 |
|  | LN845746 |
|  | LN845747 |
|  | AB451242 |
|  | BAG70056 |
|  | AB451367 |
|  | BAG70181 |
|  | AK094287 |
|  | AK122894 |
|  | BAG53786 |
|  | AK131465 |
|  | AK299310 |
|  | BAH12997 |
|  | AK314256 |
|  | BAG36922 |
|  | BC000479 |
|  | AAH00479 |
|  | BC001737 |
|  | AAH01737 |
|  | BC063408 |
|  | BC084538 |
|  | AAH84538 |
|  | BX647722 |
|  | BX648205 |
|  | CN414251 |
|  | KF836747 |
|  | AHA61679 |
|  | KF836748 |
|  | AHA61680 |
|  | KF836749 |
|  | AHA61681 |
|  | KF836750 |
|  | AHA61682 |
|  | KU177898 |
|  | ALQ33356 |
|  | KU177899 |
|  | ALQ33357 |
|  | M63167 |
|  | AAA36539 |
|  | X61037 |
|  | CAA43372 |
|  | P31749 |
|  | NC\_000078 |
|  | NM\_001165894 |
|  | NP\_001159366 |
|  | NM\_009652 |
|  | NP\_033782 |
|  | XM\_006515416 |
|  | XP\_006515479 |
|  | XM\_006515415 |
|  | XP\_006515478 |
|  | NM\_001331107 |
|  | AC124373 |
|  | AF534134 |
|  | AAN04036 |
|  | CH466549 |
|  | EDL18586 |
|  | EDL18587 |
|  | JC006884 |
|  | CDL93490 |
|  | AI843381 |
|  | AK040758 |
|  | BAC30695 |
|  | AK154936 |
|  | BAE32937 |
|  | AK196375 |
|  | AK211494 |
|  | AK214116 |
|  | AK216454 |
|  | BC066018 |
|  | AAH66018 |
|  | BC115583 |
|  | AAI15584 |
|  | KF836746 |
|  | AHA61678 |
|  | M94335 |
|  | AAA18254 |
|  | S79788 |
|  | AAB35511 |
|  | X65687 |
|  | CAA46620 |
|  | P31750 |
|  | NC\_005105 |
|  | XM\_006240631 |
|  | XP\_006240693 |
|  | XM\_008764918 |
|  | XP\_008763140 |
|  | NM\_033230 |
|  | NP\_150233 |
|  | AC\_000074 |
|  | AAHX01046747 |
|  | AC141959 |
|  | CH474034 |
|  | EDL97422 |
|  | D30040 |
|  | BAA06279 |
|  | P47196 |
|  | EU832571 |
|  | AC\_000146 |
|  | AC\_000034 |
|  | AF283830 |
|  | XP\_005267458 |
|  | XP\_005267459 |
|  | NC\_018925 |
|  | ABBA01002984 |
|  | ABBA01002985 |
|  | ABBA01002983 |
|  | AF283820 |
|  | AF283821 |
|  | AF283822 |
|  | AF283823 |
|  | AF283824 |
|  | AF283825 |
|  | AF283826 |
|  | AF283827 |
|  | AF283828 |
|  | AF283829 |
|  | ACE87663 |
|  | AABR06046383 |
|  | AAL55732 |
|  | AABR06046382 |
|  | XP\_006515480 |
|  | XP\_011534846 |
|  | XP\_011534845 |
|  | AF283818 |
|  | AF283819 |
|  | XM\_006515417 |
|  | EU832491 |
|  | XM\_005267401 |
|  | XM\_005267402 |
|  | ACE86973 |
|  | XM\_011536543 |
|  | XM\_011536544 |
|  | AMYH02029951 |
|  | AMYH02029952 |
|  | AAHY01103682 |
|  | AAHY01103681 |
|  | NT\_026437 |
|  | NW\_001838116 |
|  | NW\_925561 |
|  | AC\_000057 |
|  | AK127193 |
|  | BAG54449 |
|  | CR611982 |
|  | Q9BV07 |
|  | NT\_166318 |
|  | NW\_001030504 |
|  | Q6GSA6 |
|  | Q8BS26 |
|  | NW\_047762 |
|  | NW\_001084847 |
|  | BAD18610 |
|  | Q6ZMW5 |
|  | BG064484 |
|  | BG077899 |
|  | NW\_000055 |
|  | NT\_039551 |
|  | BE952690 |
|  | 1346401 |
|  | 485403 |
|  | 200642 |
|  | 22653384 |
|  | 26333955 |
|  | 287807 |
|  | 400144 |
|  | 41946110 |
|  | 74191998 |
|  | 81874935 |
|  | 81910973 |
|  | 119602287 |
|  | 119602288 |
|  | 119602289 |
|  | 12653417 |
|  | 12804627 |
|  | 18027298 |
|  | 190828 |
|  | 35481 |
|  | 3716606 |
|  | 3717081 |
|  | 47077445 |
|  | 54038761 |
|  | 60391226 |
|  | 74710591 |
|  | 74724882 |
|  | 133777895 |
|  | 148686639 |
|  | 148686640 |
|  | 149044040 |
|  | NT\_086807 |
|  | 110825704 |
|  | 58373279 |
|  | 631 |
|  | AAW71957 |
|  | AY781100 |
|  | CAA43371 |
|  | NC\_007319 |
|  | NM\_173986 |
|  | NP\_776411 |
|  | NW\_001494072 |
|  | Q01314 |
|  | X61036 |
|  | NC\_007116 |
|  | NW\_001513403 |
|  | XM\_692450 |
|  | XP\_697542 |
|  | NT\_039553 |
|  | XM\_192740 |
|  | XP\_192740 |
|  | 125816306 |
|  | 126007510 |
|  | 569086 |
|  | Dr.35935 |
|  | dre:569086 |
|  | 51493278 |
|  | BAC30169 |
|  | BC048179 |
|  | AK028483 |
|  | 6753033 |
|  | 6753034 |
|  | AK079866 |
|  | 190827 |
|  | 26333954 |
|  | AK149844 |
|  | BC021313 |
|  | AAH48179 |
|  | 35480 |
|  | P46737 |
|  | 400143 |
|  | 200641 |
|  | 33875493 |
|  | BAB27894 |
|  | AA588967 |
|  | AAB29006 |
|  | NT\_039706 |
|  | 51829386 |
|  | 12804626 |
|  | 3716605 |
|  | 34367364 |
|  | 3717080 |
|  | 287806 |
|  | NP\_666068 |
|  | NM\_145956 |
|  | BAE29117 |
|  | AK164578 |
|  | NC\_000086 |
|  | 34528338 |
|  | 22653383 |
|  | 4885061 |
|  | 4885060 |
|  | 18027297 |
|  | 34935858 |
|  | 15100164 |
|  | AK030736 |
|  | 15100163 |
|  | S68022 |
|  | AAH21313 |
|  | AK038919 |
|  | 485402 |
|  | 54038760 |
|  | AK011876 |
|  | 51472322 |
|  | 41946109 |
|  | NT\_039753 |
|  | BAC27105 |
|  | NP\_001318036 |
|  | NP\_001369359 |
|  | NP\_001369360 |
|  | NP\_001369361 |
|  | NP\_001369362 |
|  | NM\_001382433 |
|  | NM\_001382430 |
|  | NM\_001382432 |
|  | NM\_001382431 |

---

|  |  |
| --- | --- |
| Swiss-Prot ID | AKT1\_HUMAN |
|  | AKT1\_MOUSE |
|  | AKT1\_RAT |
|  | Q6GSA6\_MOUSE |
|  | B3KVH4\_HUMAN |
|  | B0LPE5\_HUMAN |

---

|  |  |
| --- | --- |
| Cell Localization | Cytoplasm |
|  | Nucleus |
|  | Cell membrane |

---

|  |  |
| --- | --- |
| Ensembl ID | ENSG00000142208 |
|  | ENSP00000384293.2 |
|  | ENST00000407796.6 |
|  | ENSP00000270202.4 |
|  | ENST00000349310.7 |
|  | ENSP00000450688.1 |
|  | ENST00000555528.5 |
|  | ENSMUSG00000001729 |
|  | ENSMUSP00000001780.3 |
|  | ENSMUST00000001780.9 |
|  | ENSMUSP00000122222.2 |
|  | ENSMUST00000128300.8 |
|  | ENSRNOG00000028629 |
|  | ENSRNOP00000038369.2 |
|  | ENSRNOT00000031164.3 |
|  | ENST00000554848 |
|  | ENSMUST00000128300 |
|  | ENST00000407796 |
|  | ENSMUST00000001780 |
|  | ENSP00000451166 |
|  | ENST00000349310 |
|  | ENSMUSP00000001780 |
|  | ENSP00000450688 |
|  | ENSRNOT00000031164 |
|  | ENSP00000384293 |
|  | ENSMUSP00000122222 |
|  | ENSP00000270202 |
|  | ENSRNOP00000038369 |
|  | ENST00000555528 |

---

|  |  |
| --- | --- |
| MGI ID | MGI:87986 |
|  | 87986 |
|  | 97157 |

---

|  |  |
| --- | --- |
| RGD ID | 2081 |

---

|  |  |
| --- | --- |
| Unigene ID | Rn.11422 |
|  | Hs.525622 |
|  | Mm.6645 |
|  | Bt.4843 |
|  | Dr.35935 |
|  | Hs.368861 |
|  | Hs.71816 |

---

|  |  |
| --- | --- |
| Homologene ID | 3785 |
|  | 207 |
|  | 11651 |
|  | 24185 |

---

|  |  |
| --- | --- |
| Shape | Sickle-vertex |

---

|  |  |
| --- | --- |
| IPI ID | IPI00212844 |
|  | IPI00323969 |
|  | IPI00012866 |
|  | IPI00556735 |

---

|  |  |
| --- | --- |
| Chromosome position | X A7.3 |
|  | 12 57.0 cM |
|  | 14q32.32 |
|  | 6q32 |

---

|  |  |
| --- | --- |
| Microarray ID | 100970\_at |
|  | 1368862\_at |
|  | 1375178\_at |
|  | D30040\_at |
|  | A\_43\_P12733 |
|  | NM\_033230 |
|  | 1416657\_at |
|  | 1425711\_a\_at |
|  | x65687\_s\_at |
|  | Msa.237.0\_at |
|  | A\_65\_P03244 |
|  | M94335.1 |
|  | A\_65\_P20672 |
|  | H3020C06-3 |
|  | A\_51\_P237553 |
|  | NM\_009652 |
|  | M63167 |
|  | 67939\_at |
|  | 1564\_at |
|  | 207163\_s\_at |
|  | 81268\_at |
|  | M63167\_at |
|  | rc\_aa011575\_f\_at |
|  | rc\_aa041305\_f\_at |
|  | rc\_aa044077\_f\_at |
|  | rc\_aa056416\_f\_at |
|  | rc\_aa063612\_f\_at |
|  | rc\_t03388\_f\_at |
|  | A\_23\_P14358 |
|  | I\_959380 |
|  | A\_14\_P108902 |
|  | A\_14\_P105663 |
|  | A\_14\_P138231 |
|  | A\_23\_P2960 |
|  | R083 |
|  | OH8088 |
|  | C296 |
|  | OR0248 |
|  | M640 |

---

|  |  |
| --- | --- |
| Homo sapiens Chromosome position | 14q32.32 |

---

|  |  |
| --- | --- |
| Ensembl Transcript ID | ENST00000402615 |
|  | ENST00000407796 |
|  | ENST00000349310 |

---

|  |  |
| --- | --- |
| ENSEMBL | ENSG00000142208 |

---

|  |  |
| --- | --- |
| FunctionalClass | identical protein binding |
|  | protein binding |
|  | protein kinase |
|  | protein serine/threonine kinase |
|  | protein-tyrosine kinase |
|  | receptor signaling protein serine/threonine kinase |
|  | ATP binding |
|  | kinase |
|  | nucleotide binding |
|  | sugar porter |
|  | transferase |
|  | sugar:hydrogen ion symporter |
|  | protein self binding |
|  | enzyme regulator |

---

|  |  |
| --- | --- |
| Hugo Symbol | AKT1 |

---

|  |  |
| --- | --- |
| Bos taurus Chromosome position | 21 |

---

|  |  |
| --- | --- |
| Mus musculus Chromosome position | 12 57.0 cM |

---

|  |  |
| --- | --- |
| KEGG pathway | Insulin signaling pathway |
|  | B cell receptor signaling pathway |
|  | Apoptosis |
|  | Jak-STAT signaling pathway |
|  | Focal adhesion |
|  | Tight junction |
|  | Toll-like receptor signaling pathway |
|  | MAPK signaling pathway |
|  | T cell receptor signaling pathway |
|  | Adipocytokine signaling pathway |
|  | Chronic myeloid leukemia |
|  | Colorectal cancer |
|  | Fc epsilon RI signaling pathway |
|  | Glioma |
|  | Melanoma |
|  | Pancreatic cancer |
|  | VEGF signaling pathway |
|  | mTOR signaling pathway |
|  | Endometrial cancer |
|  | ErbB signaling pathway |
|  | Non-small cell lung cancer |
|  | Prostate cancer |
|  | Renal cell carcinoma |
|  | Small cell lung cancer |

---

|  |  |
| --- | --- |
| ProteinAtlas SubcellularLocation | Main location: Nucleus; Other location: Cytoskeleton (Microtubules); Expression type: Staining; Reliability: Supportive |

---

|  |  |
| --- | --- |
| State | p |

---

|  |  |
| --- | --- |
| Rattus norvegicus Chromosome position | 6q32 |

---

|  |  |
| --- | --- |
| Ensembl Gene ID | ENSG00000142208 |

---

|  |  |
| --- | --- |
| Keyword | protein serine/threonine kinase |
|  | plasma membrane |
|  | map kinase |
|  | docking protein |
|  | cyclin |
|  | 5.4.2.- |
|  | ph domain |
|  | nucleus |
|  | map kinases |
|  | protein serine/threonine kinase activity |
|  | gtpase |
|  | cytosol |
|  | cytoplasm |
|  | MEK |
|  | protein tyrosine kinase |
|  | small monomeric GTPase |

---

|  |  |
| --- | --- |
| ProteinAtlas NormalTissue | Tissue: gall bladder; Cell type: glandular cells; Level: Strong; Expression type: Staining; Reliability: Uncertain |
|  | Tissue: hippocampus; Cell type: neuronal cells; Level: Strong; Expression type: Staining; Reliability: Uncertain |
|  | Tissue: liver; Cell type: bile duct cells; Level: Strong; Expression type: Staining; Reliability: Uncertain |
|  | Tissue: epididymis; Cell type: glandular cells; Level: Strong; Expression type: Staining; Reliability: Uncertain |
|  | Tissue: tonsil; Cell type: germinal center cells; Level: Strong; Expression type: Staining; Reliability: Uncertain |
|  | Tissue: vagina; Cell type: squamous epithelial cells; Level: Moderate; Expression type: Staining; Reliability: Uncertain |
|  | Tissue: vulva/anal skin; Cell type: epidermal cells; Level: Moderate; Expression type: Staining; Reliability: Uncertain |
|  | Tissue: spleen; Cell type: cells in red pulp; Level: Moderate; Expression type: Staining; Reliability: Uncertain |
|  | Tissue: lymph node; Cell type: germinal center cells; Level: Strong; Expression type: Staining; Reliability: Uncertain |
|  | Tissue: adrenal gland; Cell type: glandular cells; Level: Strong; Expression type: Staining; Reliability: Uncertain |
|  | Tissue: uterus |
|  | pre-menopause; Cell type: glandular cells; Level: Strong; Expression type: Staining; Reliability: Uncertain |
|  | Tissue: placenta; Cell type: decidual cells; Level: Strong; Expression type: Staining; Reliability: Uncertain |
|  | Tissue: liver; Cell type: hepatocytes; Level: Low; Expression type: APE; Reliability: High |
|  | Tissue: adrenal gland; Cell type: glandular cells; Level: High; Expression type: APE; Reliability: High |
|  | Tissue: appendix; Cell type: glandular cells; Level: Medium; Expression type: APE; Reliability: High |
|  | Tissue: appendix; Cell type: lymphoid tissue; Level: High; Expression type: APE; Reliability: High |
|  | Tissue: bone marrow; Cell type: hematopoietic cells; Level: High; Expression type: APE; Reliability: High |
|  | Tissue: breast; Cell type: glandular cells; Level: High; Expression type: APE; Reliability: High |
|  | Tissue: bronchus; Cell type: respiratory epithelial cells; Level: High; Expression type: APE; Reliability: High |
|  | Tissue: cerebellum; Cell type: cells in granular layer; Level: High; Expression type: APE; Reliability: High |
|  | Tissue: cerebellum; Cell type: cells in molecular layer; Level: High; Expression type: APE; Reliability: High |
|  | Tissue: cerebellum; Cell type: Purkinje cells; Level: Medium; Expression type: APE; Reliability: High |
|  | Tissue: cerebral cortex; Cell type: glial cells; Level: Medium; Expression type: APE; Reliability: High |
|  | Tissue: cerebral cortex; Cell type: neuronal cells; Level: Medium; Expression type: APE; Reliability: High |
|  | Tissue: cervix, uterine; Cell type: glandular cells; Level: High; Expression type: APE; Reliability: High |
|  | Tissue: cervix, uterine; Cell type: squamous epithelial cells; Level: Medium; Expression type: APE; Reliability: High |
|  | Tissue: colon; Cell type: glandular cells; Level: High; Expression type: APE; Reliability: High |
|  | Tissue: duodenum; Cell type: glandular cells; Level: High; Expression type: APE; Reliability: High |
|  | Tissue: epididymis; Cell type: glandular cells; Level: High; Expression type: APE; Reliability: High |
|  | Tissue: esophagus; Cell type: squamous epithelial cells; Level: Medium; Expression type: APE; Reliability: High |
|  | Tissue: fallopian tube; Cell type: glandular cells; Level: High; Expression type: APE; Reliability: High |
|  | Tissue: gall bladder; Cell type: glandular cells; Level: High; Expression type: APE; Reliability: High |
|  | Tissue: heart muscle; Cell type: myocytes; Level: Medium; Expression type: APE; Reliability: High |
|  | Tissue: hippocampus; Cell type: glial cells; Level: Medium; Expression type: APE; Reliability: High |
|  | Tissue: hippocampus; Cell type: neuronal cells; Level: High; Expression type: APE; Reliability: High |
|  | Tissue: kidney; Cell type: cells in glomeruli; Level: Medium; Expression type: APE; Reliability: High |
|  | Tissue: kidney; Cell type: cells in tubules; Level: Medium; Expression type: APE; Reliability: High |
|  | Tissue: lateral ventricle; Cell type: glial cells; Level: Medium; Expression type: APE; Reliability: High |
|  | Tissue: lateral ventricle; Cell type: neuronal cells; Level: Medium; Expression type: APE; Reliability: High |
|  | Tissue: liver; Cell type: bile duct cells; Level: High; Expression type: APE; Reliability: High |
|  | Tissue: lung; Cell type: macrophages; Level: Medium; Expression type: APE; Reliability: High |
|  | Tissue: lung; Cell type: pneumocytes; Level: High; Expression type: APE; Reliability: High |
|  | Tissue: lymph node; Cell type: germinal center cells; Level: High; Expression type: APE; Reliability: High |
|  | Tissue: lymph node; Cell type: non-germinal center cells; Level: High; Expression type: APE; Reliability: High |
|  | Tissue: nasopharynx; Cell type: respiratory epithelial cells; Level: High; Expression type: APE; Reliability: High |
|  | Tissue: oral mucosa; Cell type: squamous epithelial cells; Level: Medium; Expression type: APE; Reliability: High |
|  | Tissue: ovary; Cell type: follicle cells; Level: High; Expression type: APE; Reliability: High |
|  | Tissue: ovary; Cell type: ovarian stroma cells; Level: High; Expression type: APE; Reliability: High |
|  | Tissue: pancreas; Cell type: exocrine glandular cells; Level: High; Expression type: APE; Reliability: High |
|  | Tissue: pancreas; Cell type: islets of Langerhans; Level: High; Expression type: APE; Reliability: High |
|  | Tissue: parathyroid gland; Cell type: glandular cells; Level: Medium; Expression type: APE; Reliability: High |
|  | Tissue: placenta; Cell type: decidual cells; Level: High; Expression type: APE; Reliability: High |
|  | Tissue: placenta; Cell type: trophoblastic cells; Level: High; Expression type: APE; Reliability: High |
|  | Tissue: prostate; Cell type: glandular cells; Level: Medium; Expression type: APE; Reliability: High |
|  | Tissue: rectum; Cell type: glandular cells; Level: High; Expression type: APE; Reliability: High |
|  | Tissue: salivary gland; Cell type: glandular cells; Level: Medium; Expression type: APE; Reliability: High |
|  | Tissue: seminal vesicle; Cell type: glandular cells; Level: High; Expression type: APE; Reliability: High |
|  | Tissue: skeletal muscle; Cell type: myocytes; Level: Low; Expression type: APE; Reliability: High |
|  | Tissue: skin; Cell type: epidermal cells; Level: Medium; Expression type: APE; Reliability: High |
|  | Tissue: small intestine; Cell type: glandular cells; Level: Medium; Expression type: APE; Reliability: High |
|  | Tissue: smooth muscle; Cell type: smooth muscle cells; Level: Medium; Expression type: APE; Reliability: High |
|  | Tissue: spleen; Cell type: cells in red pulp; Level: Medium; Expression type: APE; Reliability: High |
|  | Tissue: spleen; Cell type: cells in white pulp; Level: Medium; Expression type: APE; Reliability: High |
|  | Tissue: stomach, lower; Cell type: glandular cells; Level: Medium; Expression type: APE; Reliability: High |
|  | Tissue: stomach, upper; Cell type: glandular cells; Level: Medium; Expression type: APE; Reliability: High |
|  | Tissue: testis; Cell type: cells in seminiferus ducts; Level: High; Expression type: APE; Reliability: High |
|  | Tissue: testis; Cell type: Leydig cells; Level: Medium; Expression type: APE; Reliability: High |
|  | Tissue: thyroid gland; Cell type: glandular cells; Level: Medium; Expression type: APE; Reliability: High |
|  | Tissue: tonsil; Cell type: germinal center cells; Level: High; Expression type: APE; Reliability: High |
|  | Tissue: tonsil; Cell type: non-germinal center cells; Level: High; Expression type: APE; Reliability: High |
|  | Tissue: tonsil; Cell type: squamous epithelial cells; Level: Medium; Expression type: APE; Reliability: High |
|  | Tissue: urinary bladder; Cell type: urothelial cells; Level: High; Expression type: APE; Reliability: High |
|  | Tissue: uterus, post-menopause; Cell type: cells in endometrial stroma; Level: High; Expression type: APE; Reliability: High |
|  | Tissue: uterus, post-menopause; Cell type: glandular cells; Level: High; Expression type: APE; Reliability: High |
|  | Tissue: uterus, pre-menopause; Cell type: cells in endometrial stroma; Level: High; Expression type: APE; Reliability: High |
|  | Tissue: uterus, pre-menopause; Cell type: glandular cells; Level: High; Expression type: APE; Reliability: High |
|  | Tissue: vagina; Cell type: squamous epithelial cells; Level: Medium; Expression type: APE; Reliability: High |
|  | Tissue: vulva/anal skin; Cell type: epidermal cells; Level: Medium; Expression type: APE; Reliability: High |

---

|  |  |
| --- | --- |
| LocatorString | gscquery:curcumin+prostate+cancer |
|  | pmquery:curcumin+prostate+cancer |
|  | pmquery:curcumin prostate cancer |
|  | pmquery:egfr+transactivation |

---

|  |  |
| --- | --- |
| EnzymeConsortium | 2.7.1.37 |

---
